# Effectiveness of wastewater treatment systems in removing microbial agents: a systematic review

**DOI:** 10.1186/s12992-020-0546-y

**Published:** 2020-02-03

**Authors:** Zahra Aghalari, Hans-Uwe Dahms, Mika Sillanpää, Juan Eduardo Sosa-Hernandez, Roberto Parra-Saldívar

**Affiliations:** 1 Faculty of Public Health, Gonabad University of Medical Sciences, Gonabad, Iran; 2 Department of Biomedical Science and Environmental Biology, Kaohsiung Medical University, Kaohsiung, 80708 Taiwan, Republic of China; 3 Research Center for Environmental Medicine, Kaohsiung Medical University, Kaohsiung, 80708 Taiwan, Republic of China; 4 Department of Marine Biotechnology and Resources, National Sun Yat-sen University, Kaohsiung, 80424 Taiwan, Republic of China; 5 Department of Civil and Environmental Engineering, Florida International University, Miami, FL USA; 6 Tecnologico de Monterrey, School of Engineering and Sciences, Campus Monterrey, Ave. Eugenio Garza Sada 2501, CP 64849 Monterrey, Nuevo Leon Mexico

**Keywords:** Systematic analysis, Wastewater, Treatment, Microbial agents, Environmental health, Articles, Journals

## Abstract

**Background:**

Due to unrestricted entry of wastewater into the environment and the transportation of microbial contaminants to humans and organisms, environmental protection requires the use of appropriate purification systems with high removal efficiency for microbial agents are needed. The purpose of this study was to determine the efficacy of current wastewater treatment systems in removing microbes and their contaminants.

**Methods:**

A systematic review was conducted for all articles published in 5 Iranian environmental health journals in 11 years. The data were collected according to the inclusion and exclusion criteria and by searching the relevant keywords in the articles published during the years (2008–2018), with emphasis on the efficacy of wastewater treatment systems in removing microbial agents. Qualitative data were collected using a preferred reporting items for systematic reviews and meta-analyzes (PRISMA) standard checklist. After confirming the quality of the articles, information such as the name of the first author and the year of publication of the research, the type of study, the number of samples, the type of purification, the type of microbial agents and the rate of removal of microbial agents were entered into the checklist. Also the removal rates of the microbial agents mentioned in the studies were compared with united states environmental protection agency (US-EPA) standards.

**Results:**

In this study, 1468 articles retrieved from 118 issues of 5 environmental health journals were reviewed. After reviewing the quality of the articles in accordance with the research objectives, 14 articles were included in the study that were published between 2010 and 2018. In most studies, two main indicators *Total coliforms* and *Fecal coliforms* in wastewater were investigated. Removing fungi and viral contamination from wastewater was not found in any of the 14 studies. Different systems (activated sludge, stabilization ponds, wetlands, and low and medium pressure UV disinfection systems were used to remove microbial agents in these studies. Most articles used active sludge systems to remove *Total coliforms* and *Fecal coliforms*, which in some cases were not within the US-EPA standard. The removal of *Cysts* and *Parasitic eggs* was only reporte from stabilization pond systems (SPS) where removal efficiency was found in accordance with US-EPA standards.

**Conclusions:**

Different types of activated sludge systems have higher efficacy to remove microbial agents and are more effective than other mentioned systems in removing the main indicators of sewage contamination including *Total coliforms* and *Fecal coliforms*. However, inappropriate operation, maintenance and inadequate handling of activated sludge can also reduce its efficiency and reduce the removal of microbial agents, which was reported in some studies. Therefore, it is recommended to conduct research on how to improve the operation, maintenance, and proper management of activated sludge systems to transfer knowledge to users of sludge systems and prevent further health issues related to microbial agents.

## Introduction

Due to hazardous impacts of municipal, industrial and hospital wastewater on water, soil, air and agricultural products, wastewater treatment and the proper disposal of the sludge produced are indispensable from an environmental safety point of view [1, 2]. Economically, effective wastewater treatment has important effects on saving water, and preventing unnecessary water losses [3]. In arid and semiarid countries such as Iran, the water demand has increased and annual rainfall is low also in regions of North Africa, Southern Europe, and in large countries such as Australia and the United States. Consequently, reuse of sewage is the most sustainable and long-term solution to the problem of water scarcity [4, 5]. In the next 30 years, the world’s population will increase by more than double. Due to population growth, the amount of water available in 1960 was reduced to 3300 cubic meters and in 1995 it was reduced to 1250 cubic meters. This trend is projected to decrease to 650 cubic meters worldwide by 2025 [6]. Due to this water shortage crisis, water from wastewater treatment need to be reused increasingly in the near future [6]. Wastewater reuse requires treatment and application of appropriate wastewater treatment systems [7]. In recent years, increased research has been done on wastewater treatment using simple, low-cost, easy-to-use methods in developing countries [8, 9]. Systems and processes such as activated sludge, aerated lagoons, stabilization ponds, natural and synthetic wetlands, trickling filters, rotating biological contactors (RBCs) have been used for wastewater treatment and removal of physical, chemical and biological contaminants [10, 11]. Among different contaminants of wastewater, microbial agents becoming increasingly important and their removal efficiency should be reported in different wastewater treatment systems [12, 13]. Biological contaminants in wastewater are different types of bacteria (*Fecal coliforms* and *Escherichia coli*, *Salmonella*, *Shigella*, *Vibrio cholerae*), diverse *Parasite cysts* and *eggs*, viruses and fungi. All of them can be hazardous to environmental and human health depending on the type and amount [14, 15]. For example, bacteria in wastewater cause cholera, typhoid fever, and tuberculosis, viruses can cause hepatitis, and protozoa can cause dysentery [16, 17]. Many microbial agents attached to suspended solids in wastewater if inadequately treated and wastewater discharge into the environment, such as river water, green space, and crops, put humans and aquatic organisms at risk [18, 19]. Therefore, utilization of appropriate wastewater treatment systems tailored to a variety of microbial agents is essential to achieve as complete as possible elimination of biological agents. For example, in the study of Sharafi et al., (2015) with the aim of determining the removal efficiency of parasites from wastewater using a wetland system, the removal rates of protozoan cysts and *Parasite eggs* were 99.7 and 100%, respectively [20]. Okoh, et.al. (2010) reported that activated sludge processes, oxidation pools, activated carbon filtration, lime and chlorination coagulation eliminated removed 50–90% of wastewater viruses [21]. Wastewater from wastewater treatment plants, is used in Iran without restrictions and controls like in many other countries. Therefore, it is necessary to employ proper sewage treatment systems, before water can be publicly used such as for irrigation. This study is focusing on the efficacy of different wastewater treatment systems in removing microbial agents.

## Methods

### Study protocol

This systematic review study was carried out to determine the efficacy of wastewater treatment systems in the removal of microbial agents (bacteria, parasites, viruses, and fungi) by searching all articles published in 5 Iranian Journals of Environmental Health. The data were collected by referring to the specialized site of each journal, from the beginning of 2008 to the latest issue of 2018. Reviewed journals included; Iranian Journal of Health and Environment (IJHE), Journal of Environmental Health Engineering (JEHE), Journal of Research in Environmental Health (JREH), and two English-language journals, Environmental Health Engineering and Management Journal (EHEMJ), Journal of Environmental Health Science and Engineering (JEHSE).

### Search strategy

Inquired information was collected by searching for keywords on the sites of Iranian specialty health journal. Key words included; ‘waste water’ OR ‘waste-water’ OR ‘wastewater treatment’ OR ‘effluent’ OR ‘sewage’ OR ‘sewage treatment’ OR ‘sewage disposal’ OR ‘wastewater disposal’ AND ‘treat’ OR ‘remove’ AND ‘microb’ AND ‘pathogen’ AND ‘bacteria’ AND ‘virus’ AND ‘parasite’ AND ‘FCs’ OR *‘Fecal coliforms*’ AND ‘Iran’.

A manual search was performed by checking all published articles. This way, the abstracts of all published articles were reviewed over the period of 11 years between 2008 and 2018.

### Inclusion criteria

Inclusion criteria for this study included the year of publication, type of wastewater samples (municipal wastewater, domestic wastewater, hospital wastewater), number of samples (more than 5 wastewater samples), treatment procedures (different types), state the required and mention the type of purification (type of treatment, type of microbial agents, amount or percentage of microbial agents removed).

### Exclusion criteria

Exclusion criteria for this study were: lack of access to the full article, inappropriate subject matter, inadequacy of the method of treatment and purification, lack of expression of the type of microbial agents removed, review studies, and letters to the editor.

### Quality assessment articles

This study is based on standard checklist PRISMA (Preferred Reporting Items for Systematic Reviews and Meta-analyzes). The US-based National Institute of Health Quality Assessment Tool for Observational Cohort and Cross-Sectional Studies [22] for qualitative studies was used. This checklist is made based on the following criteria: Yes, No, cannot determine, Not applicable, and Not reported. It has eliminated the scoring problems. The checklist included 14 questions that were used for research purposes, samples, inclusion and exclusion criteria, findings, results and publication period of each of the 14 articles (Table 1).

**Table 1:** Check list of quality assessment tool for observational cohort and cross-sectional studies (Ref. [22])

Criteria	
1. Was the research question or objective in this paper clearly stated?	
2. Was the study population clearly specified and defined?	
3. Was the participation rate of eligible persons at least 50%?	
4. Were all the subjects selected or recruited from the same or similar populations (including the same time period)? Were inclusion and exclusion criteria for being in the study prespecified and applied uniformly to all participants?	
5. Was a sample size justification, power description, or variance and effect estimates provided?	
6. For the analyses in this paper, were the exposure(s) of interest measured prior to the outcome(s) being measured?	
7. Was the timeframe sufficient so that one could reasonably expect to see an association between exposure and outcome if it existed?	
8. For exposures that can vary in amount or level, did the study examine different levels of the exposure as related to the outcome (e.g., categories of exposure, or exposure measured as continuous variable)?	
9. Were the exposure measures (independent variables) clearly defined, valid, reliable, and implemented consistently across all study participants?	
10. Was the exposure(s) assessed more than once over time?	
11. Were the outcome measures (dependent variables) clearly defined, valid, reliable, and implemented consistently across all study participants?	
12. Were the outcome assessors blinded to the exposure status of participants?	
13. Was loss to follow-up after baseline 20% or less?	
14. Were key potential confounding variables measured and adjusted statistically for their impact on the relationship between exposure(s) and outcome(s)?	

### Extract information from articles

In order to extract information, all articles were evaluated independently by two reviewers based on inclusion and exclusion criteria. Both reviewers eventually summarized the information and in cases where the information was inconsistent a third reviewer’s comments was used. The information extracted from the articles was included in the researcher’s checklist for qualitative approval and used in other prior author studies of this paper [23–25]. The checklist included the name of the first author, the year of publication of the research, the type of study, the number of samples, the type of purification, the type of microbial agents and the rate of microbial removal. Additionally, the removal rates of the microbial agents mentioned in the studies were compared with US-EPA standards [26, 27] (Table 2).

**Table 2:** Removal of microbial agents in treated wastewater according to US-EPA standards (Ref. [26, 27])

Parameter	Standard
*Total coliforms*	1000 ^a^MPN/100 mL
*Salmonella*	Not detected/50 g of final product
*Escherichia coli*	< 100 ^a^MPN per gram (dry weight)
*Fecal coliforms*	< 1000 ^a^MPN per gram (dry weight)
*Enteric viruses*	< 1 PFU per 4 g total dry solids
*Helminth eggs (Ascaris sp. and Taenia sp.)*	< 1 per 4 g total dry solids

^a^*MPN* Most Probable Number

## Findings

### Search results

**Fig. 1:**
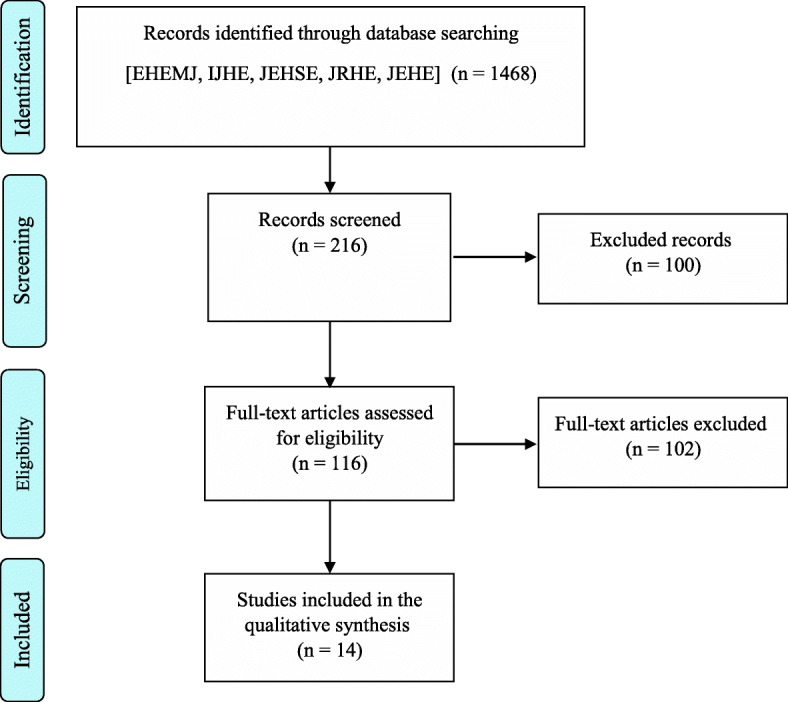
Flowchart describing the study design

In this study, 1468 articles related to 118 issues of 5 environmental health journals were reviewed. In the first phase of the search process, 216 articles on wastewater treatment were identified. Then, 196 inappropriate and irrelevant articles were excluded for the purpose of the study. Finally, after reviewing the information and quality of the articles, 14 articles were eligible for systematic review (Fig. 1).

### Descriptive results of articles

**Fig. 2:**
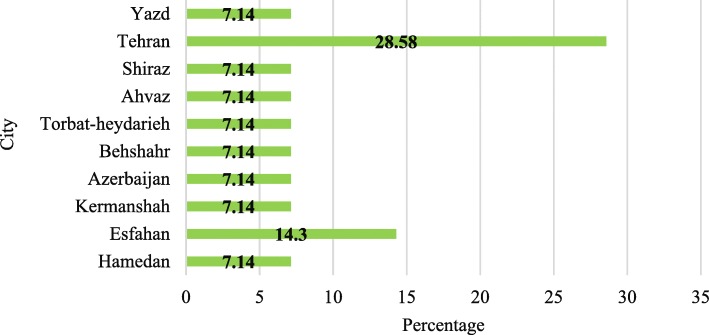
Cities selected for wastewater sampling in 14 articles

Of the 14 articles reviewed, the largest number of articles (9 articles; 64.2%) were published between 2014 and 2018. Most of the experiments were carried out on wastewater samples in Tehran (28.58%). In total, studies were conducted in 10 cities of Iran (Fig. 2).

**Fig. 3:**
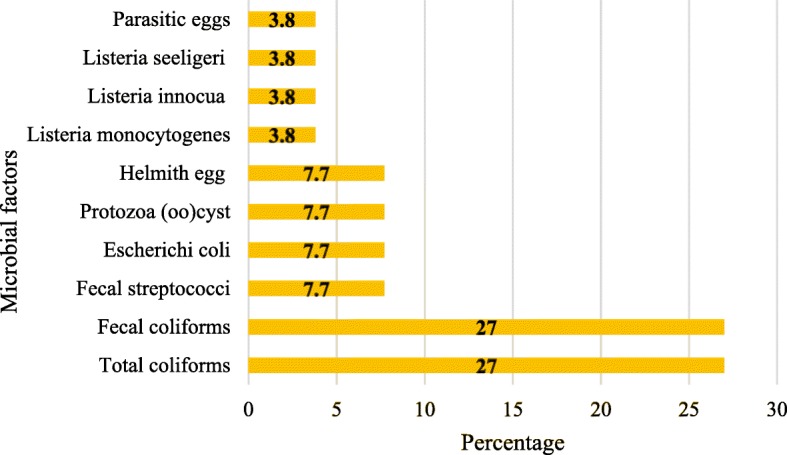
Types of microbial agents removed in wastewater based on the articles

Concerning the type of microbial agents, it was found that a total of 14 articles have eliminated types of bacteria and parasites from municipal, hospital and industrial wastewater (Fig. 3). In 11 articles, two main microbial indices (*Total coliforms* and *Fecal coliforms*) were used as bioindicators to evaluate the efficacy of the wastewater treatment systems (Fig. 3).

### Quality assessment of articles

The qualitative results of the articles showed that most of the studies were of good quality but in many articles the method of determination of sample size (Q5) was not specified. In the articles, participation rate of eligible persons, inclusion and exclusion criteria, exposure (s) were evaluated more than once, and blinding of participant exposure status was not relevant and not applicable (Q10, Q4, Q3 and Q12) (Table 3).

**Table 3:** Quality of studies using the quality assessment of the NIH for cohort and cross-sectional studies

Author/Year/ Ref	Q1	Q2	Q3	Q4	Q5	Q6	Q7	Q8	Q9	Q10	Q11	Q12	Q13	Q14
Hashemi et al., 2010 [28]	✓	✓	NA	NA	✓	✓	✓	✓	✓	NA	✓	NA	✓	✓
Banejad et al., 2010 [29]	✓	✓	NA	NA	✓	✓	✓	✓	✓	NA	✓	NA	✓	✓
Derayat et al., 2011 [30]	✓	✓	NA	NA	✓	✓	✓	✓	✓	NA	✓	NA	✓	✓
Baghapour et al., 2013 [31]	✓	✓	NA	NA	×	✓	✓	✓	✓	NA	✓	NA	✓	✓
Safari et al., 2013 [32]	✓	✓	NA	NA	×	✓	✓	✓	✓	NA	✓	NA	✓	✓
Navidjouy et al., 2014 [33]	✓	✓	NA	NA	×	✓	✓	✓	✓	NA	✓	NA	✓	✓
Karimi et al., 2014 [34]	✓	✓	NA	NA	×	✓	✓	✓	✓	NA	✓	NA	✓	✓
Aslani et al., 2014 [35]	✓	✓	NA	NA	×	✓	✓	✓	✓	NA	✓	NA	✓	✓
Jamshidi et al., 2014 [36]	✓	✓	NA	NA	×	✓	✓	✓	✓	NA	✓	NA	✓	✓
Nahavandi et al., 2015 [37]	✓	✓	NA	NA	×	✓	✓	✓	✓	NA	✓	NA	✓	✓
Ghoreishi et al., 2016 [38]	✓	✓	NA	NA	×	✓	✓	✓	✓	NA	✓	NA	✓	✓
Mollaie Tavani et al., 2017 [39]	✓	✓	NA	NA	×	✓	✓	✓	✓	NA	✓	NA	✓	✓
Sasani et al., 2017 [40]	✓	✓	NA	NA	NA	✓	✓	✓	✓	NA	✓	NA	✓	✓
Choopan et al., 2018 [41]	✓	✓	NA	NA	✓	✓	✓	✓	✓	NA	✓	NA	✓	✓

^*^Cases that were followed in the articles were marked✓ and those that were not followed were marked×. Items that were not executable were also identified by the word “*NA*” not applicable

### Article features

Articles on the efficacy of a variety of purification systems for the removal of microbial agents were published between 2010 and 2018. All studies don in the laboratory. The largest sample size was related to Derayat et al., 2011 [30] in Kermanshah with 120 wastewater samples. Wastewater studies were carried out in different cities of North, East, West and Central Iran. Most studies have investigated bacterial factors in wastewater and the efficacy of removing fungi and viral contamination in wastewater was not found in any study (Table 4). In most articles, the type of sewage treatment system was activated sludge. For example were the removal rates of microbial agents in wastewater investigated in the study by Derayat et al., 2011 [30], Baghapour et al., 2013 [31] and Nahavandi et al., 2015 [37] on Conventional Activated Sludge, Ghoreishi et al., 2016 [38] on extended aeration activated sludge (Table 4).

**Table 4:** Information from articles on the efficacy of different wastewater treatment systems to remove microbial agents

Author/Year/Ref	Sample Size	Type of samples/City	Types of wastewater treatment systems	Microbial agent	Microbial agent removal rate	Compliance with US-EPA Standard (Ref. [26, 27])
Hashemi et al., 2010 [28]	17	Municipal wastewater/Esfahan	UV disinfection system including low pressure (LP)	*Total coliforms*	1000 MPN/100 mL	Yes
				*Fecal coliforms*	400 MPN/100 mL	Yes
				*Fecal streptococci*	400 MPN/100 mL	Yes
			UV disinfection system including medium pressure (MP)	*Total coliforms*	1000 MPN/100 mL	Yes
				*Fecal coliforms*	400 MPN/100 mL	Yes
				*Fecal streptococci*	400 MPN/100 mL	Yes
			LP + MP	*Total coliforms*	1000 MPN/100 mL	Yes
				*Fecal coliforms*	400 MPN/100 mL	Yes
				*Fecal streptococci*	400 MPN/100 mL	Yes
Banejad et al., 2010 [29]	12	Domestic wastewater/Hamedan	Flocculation and coagulation with moringa peregrina seeds	*Total coliforms*	97%	Yes
				*Escherichia coli*	97%	Yes
Derayat et al., 2011 [30]	120	Municipal wastewater/Kermanshah and Gilangharb	Conventional activated sludge	*Cysts*	97.5%	Yes
				*Parasite eggs*	98.3%	Yes
			Stabilization pond systems	*Cysts*	100%	Yes
				*Parasite eggs*	100%	Yes
Baghapour et al., 2013 [31]	64	Municipal wastewater/Shiraz	Activated sludge	*Total coliforms*	1291.11 ± 1165.88 MPN/100 mL	No
				*Fecal coliforms*	675.22 ± 1008.21 MPN/100 mL	No
				*Helminth egg*	73.61 ± 96.125 N/L	No
Safari et al., 2013 [32]	7	Municipal wastewater/Shahrak Gharb Tehran	Two-stage fluidized bed reactor (FBR)	*Fecal coliforms (without chlorine addition)*	35–75%	No
				*Fecal coliforms (with the chlorine addition)*	67–97%	No
Navidjouy et al., 2014 [33]	8	Municipal treatment plants and slaughterhouse treatment plants/Tehran	Activated sludge	*Helminth egg*	94.8–95.7%	Yes
				*Protozoa (oo)cyst*	79.3–85.8%	No
Karimi et al., 2014 [34]	100	Municipal wastewater/Yazd	Wetlands	*Fecal coliforms*	1.13 × 10^14^ MPN/100	No
				*Escherichia coli*	5.03 × 10^12^ MPN/100 mL	No
Aslani et al., 2014 [35]	9	Municipal wastewater/North of Tehran	Activated sludge effluent	*Fecal coliforms*	5.5 ± 05 MPN/100 mL	No
Jamshidi et al., 2014 [36]	70 Lit	Domestic wastewater/Tehran	Anaerobic baffled reactor (ABR) followed by Bio-rack wetland planted with Phragmites sp. and Typha sp.	*Total coliforms*	99%	Yes
Nahavandi et al., 2015 [37]	8	Municipal treatment plants and slaughterhouse treatment plants/Tehran	Activated sludge	*Helminth egg*	94.8–95.7%	Yes
				*Protozoa (oo)cyst*	79.3–85.8%	No
Ghoreishi et al., 2016 [38]	9	Municipal wastewater/Azerbaijan Province	Extended aeration activated sludge/Tabriz	*Total coliforms*	2.17 × 10^5^ MPN/100 mL	No
			Extended aeration activated sludge/Marand	*Total coliforms*	1.34 × 10^6^ MPN/100 mL	No
			Extended aeration activated sludge/Jolfa	*Total coliforms*	1.82 × 10^6^ MPN/100 mL	No
			Activated sludge/Ajabshir	*Total coliforms*	4.53 × 10^5^ MPN/100 mL	No
			Extended aeration activated sludge/Ahar	*Total coliforms*	2.25 × 10^3^ MPN/100 mL	No
			Extended aeration activated sludge/Mianeh	*Total coliforms*	3.93 × 10^3^ MPN/100 mL	No
			Conventional activated sludge/Maragheh	*Total coliforms*	3.02 × 10^4^ MPN/100 mL	No
			SBR/Bostanabad	*Total coliforms*	1.09 × 10^6^ MPN/100 mL	No
			Extended aeration activated sludge/Sarab	*Total coliforms*	2.02 × 10^3^ MPN/100 mL	No
Mollaie Tavani et al., 2017 [39]	16	Hospital wastewater/Behshahr	Conventional activated sludge	*Total coliforms*	46 MPN/100 mL	Yes
				*Fecal coliforms*	4.75 MPN/100 mL	Yes
Sasani et al., 2017 [40]	36	Municipal wastewater/Ahvaz	Conventional activated sludge	*Total coliforms*	7.8 × 10^12^ MPN/100 mL	No
Choopan et al., 2018 [41]	45	Municipal wastewater/Torbat-heydarieh	Activated sludge	*Fecal coliforms*	200 MPN/100 mL	Yes

*In compliance with the US-EPA standard, the results of studies within the US-EPA standard range were marked with Yes and the results of studies that exceeded the US-EPA standard were marked with No.

Evaluation of the removal of microbial agents in accordance with US-EPA standards showed that in some articles the removal of *Total coliforms* and *Fecal coliforms* was not within acceptable ranges. For example, in the study of Ghoreishi et al., 2016 [38], although several different systems were used to remove Total coliforms, eimination efficiency never reached US-EPA standards. Moreover, the activated sludge process did not have the efficiency to remove *Parasitic eggs* as reported in the study by Nahavandi et al., 2015 [37] (Table 4).

## Discussion

Examination of microbial removal rates in the study of Ghoreishi et al., 2016 [38] that none of the *Total Coliforms* removal was US-EPA standard although both extended aeration activated sludge and conventional activated sludge systems were used to remove *Total coliforms*. The US-EPA standard for *Total coliforms* removal is 1000 MPN/100 mL, and wastewater showing this amount of *Total coliforms* is capable of being discharged into the receiving waters [26, 27]. A study by Paiva et al., 2015 on domestic wastewater in tropical Brazil also showed that removal of *Total coliforms* through the use of activated sludge was not a desirable remediation method [42]. The reason for the poor performance of activated sludge to remove *Total coliforms* can be attributed to factors such as management problems and operation of the activated sludge system, which results in the production of bulk waste and sludge. This problem is one of the most important disadvantages of activated sludge systems and should be addressed once a month by experienced staff and monitoring experts to correct it. Overall, different activated sludge systems are the best choice for this type of wastewater due to the amount of municipal wastewater pollutants because of high purification efficiency to reduce biochemical oxygen demand (BOD_5_) [43, 44].

Removal of *Cysts* and *Parasitic eggs* in the study of Derayat et al., (2011), which used stabilization pond systems, was reported as being in accordance with US-EPA standards [30]. A study by Amahmid et al. (2002) aimed at the treatment of municipal wastewater with a stabilized pond system in Morocco showing that *Cyst* and *Parasitic egg* removal efficiency was 100% and that the pond system showed a proper performance [45]. A large number of stabilized pond systems were been constructed and used in countries such as the United States, New Zealand, India, Pakistan, Jordan and Thailand [3]. In Iran, a number of these systems were constructed for the treatment of wastewater in Arak, Gilan West and Isfahan [46]. Stabilization ponds have a high acceptability due to their simplicity of operation, and lack of mechanical and electrical equipment compared to other sewage treatment systems, their high efficiency in removing pathogenic organisms [47]. A major drawback for stabilization ponds is the need for extensive land, the low quality of effluents due to the presence of algae, and odor production that limits the use of this type of treatment system near habitated areas. To improve the quality of resulting effluents, chemical compounds need to be consolidated, such as by coagulation and the application of microstrainers, stabilization ponds and rock filters [47, 48].

As for wetlands by Karimi et al. (2014) on *Fecal coliforms*, *Escherichia coli* and *Fecal streptococci* show that wetlands did not perform well to remove microbial agents (removal rate for *Fecal coliforms* 1.13 × 1014 MPN/100 mL and *Escherichia coli* 5.03 × 1012 MPN/100 mL) [34]. In a study by Decamp et al. (2000), the mean removal of *Escherichia coli* through the wetland was 41 to 72% at the in situ scale and 96.6 to 98.9% at the experimental scale [49]. In the study of Evanson et al. (2006), *Fecal coliforms* removal rate was 82.7 to 95.99% [50]. Removal of *Total coliforms* and *Fecal coliforms* in the wetlands is done by various biological factors such as nematodes, protozoa, bacterial activity, bacteriophage production, chemical factors, oxidation reactions, bacterial uptake and toxicity [51] and the interference in each of these (microbial communities) will affect the rate of removal of *Total coliforms* and other microbial agents. Removal of pathogens such as *Escherichia coli* and *Cryptosporidium* was also performed in wetlands but is often not in compliance with environmental standards [52]. In addition, although wetlands are economical and widely used in wastewater treatment systems because of easy to operate, maintain, and operate at a low price [53–55], but they don’t seem to be a good option for removing all of the microbial agents.

In a study by Hashemi, et.al. (2010) on UV disinfection system included low pressure (LP) and UV disinfection system including medium pressure (MP) to remove *Total coliforms*, *Fecal coliforms* and *Fecal streptococci.* All investigated microbial agents were completely eliminated [28]. However, it was reported that the direct disinfection of secondary effluents with LP and MP systems and even their integration due to high concentrations of suspended solids was not possible. Therefore, disinfection of wastewater with UV irradiation requires higher effluent quality through improved system utilization or application of an advanced treatment plant prior to disinfection [28]. In 1988, about 300 and in 2004 about 4300 sewage treatment plants in the United States, (that are more than 20% of filtration plants) used a UV system for wastewater disinfection. The number of wastewater treatment plants having UV systems has increased in the US, Europe and East Asia. This trend is expected to expand further in the coming decades. Although the use of UV radiation for wastewater disinfection has many potential advantages, it also has disadvantages in terms of cost, lamp deposition, and the possible reactivation of targeted pathogenic microorganisms after treatment [56]. Wastewater treatment professionals should therefore be aware of new replacement processes and perform pilot scale assessments prior to changing treatment processes.

One of the strengths of this study is addressing the efficacy of wastewater treatment systems by comparing the removal efficiency of various microbial agents that have received little attention as yet. In most studies, only one type of system for removing different physical, chemical and microbial contaminants in a single type of wastewater was investigated and it was not possible to compare the removal efficiency of microbial agents. One of the limitations of this study was the lack of reviewing published articles on wastewater treatment systems in other than the 5 Iranian journals. This limitation, however, might be negligible because the research on wastewater treatment was done by environmental health professionals. Therefore, most studies in this area are published in specialized environmental health journals.

## Conclusion

Different types of activated sludge systems have better efficacy to remove microbial agents and are more effective than other systems in removing the main indicators of sewage contamination including *Total coliforms* and *Fecal coliforms*. However, inappropriate operation, maintenance and inadequate handling of activated sludge can also reduce the efficiency of microbial agent removal, which has been reported in some studies. Therefore, it is recommended to conduct research on how to increase the operation, maintenance and proper management of activated sludge systems and provide the results to utility personnel responsible to work with this system in order to correct the system quality output in a timely manner. In future research, it is recommended that employed treatment methods integrate two or more purification systems, which then could more effectively remove microbial agents. Additionally, the reports of removal efficiency should include each of the indicated microbes so that health and environmental professionals can make better decisions about using the systems or prevent future eventualities.

## Data Availability

Not applicable.

## Abbreviations

ABR: Anaerobic baffled reactor
BOD_5_: Biochemical Oxygen Demand
EHEMJ: Environmental Health Engineering and Management Journal
FBR: Fluidized Bed Reactor
IJHE: Iranian Journal of Health and Environment
JEHE: Journal of Environmental Health Engineering
JEHSE: Journal of Environmental Health Science and Engineering
JREH: Journal of Research in Environmental Health
LP: Low pressure
MP: Medium pressure
MPN: Most Probable Number
PRISMA: Preferred Reporting Items for Systematic Reviews and Meta-analyzes
RBCs: Rotating Biological Contactors
SPS: Stabilization Pond Systems
US-EPA: United States Environmental Protection Agency
UV: Ultraviolet
